# Trauma Informed Delinquency Interventions for Native Children

**DOI:** 10.1017/jme.2023.16

**Published:** 2022

**Authors:** Addie C. Rolnick, Patricia Sekaquaptewa

**Affiliations:** 1: UNIVERSITY OF NEVADA, LAS VEGAS, NV, USA; 2: UNIVERSITY OF ALASKA FAIRBANKS, FAIRBANKS, AK, USA

**Keywords:** Trauma, Indigenous, Native, Children, Delinquency

## Abstract

Recognizing the links between childhood trauma and delinquency, many juvenile delinquency systems now emphasize trauma-informed care. This commentary examines established and emerging research on childhood trauma among American Indian and Alaska Native children and contrasts the development and implementation of “trauma-informed” approaches in state and tribal juvenile systems. It identifies three key innovations present in tribal models and calls for further research to identify best practices that work for Native children and tribal communities.

Widespread recognition that trauma can lead to youth involvement in the juvenile delinquency system has spurred increasing interest in “trauma-informed” delinquency interventions and responses. This is so in large part because behaviors associated with trauma, resistance, and recovery have been labeled “delinquent acts” or “status offenses” that lead to court involvement. In many cases, juvenile delinquency is nothing more than the criminalization of traumatic responses. Accepting this link, juvenile justice policy professionals have made trauma-informed approaches a central concern as they seek to divert children away from systems that criminalize and incorporate restorative approaches to wrongdoing.

American Indian and Alaska Native (AI/AN)^1^ children are especially likely to experience traumatic events and life circumstances.^2^ Researchers have documented a relationship between these experiences and higher likelihood of involvement in the delinquency system.^3^ In the most direct relationship, children responding to a traumatic event may become involved in the system because their survival or self-protective behaviors are in themselves lawbreaking. For example, a child who runs away to escape sexual abuse at home is breaking the law and may be referred to court. Similarly, a child who uses alcohol or drugs to cope with traumatic experiences may be referred to court for underage drinking, possession of illegal substances, public drunkenness, or driving under the influence. Here, the lawbreaking behavior is a direct response to trauma.

Compared to other groups, Native youth are especially likely to enter the system for status offenses^4^ and offenses associated with family conflict and alcohol and substance abuse — offenses that do not harm others and can be easily characterized as survival behaviors.^5^ This, along with the widespread experience of traumatic victimization among Native youth, suggests that trauma is perhaps the most important driver of delinquency for them. Girls (Native and non-Native) show a similar pattern of system involvement for survival behaviors.^6^ This suggests that Native girls might be especially at risk for this kind of trauma-delinquency pipeline.

Compared to other groups, Native youth are especially likely to enter the system for status offenses and offenses associated with family conflict and alcohol and substance abuse — offenses that do not harm others and can be easily characterized as survival behaviors.

Less directly, children who have survived trauma may become perpetrators of violence, or act out in ways that harm people or property, leading to their involvement in the delinquency system. Personal experiences with assaultive trauma are especially likely to result in post-traumatic stress disorder (PTSD) and later risk of psychiatric disorders.^7^ PTSD and associated mental health and substance abuse problems can lead youth to engage in a range of behaviors that may harm others, from stealing to fighting to perpetrating sexual abuse. In this scenario, the behavior may not be a direct defensive or survival response to trauma, but it is a response to the long-term impact of that trauma. While Native youth are over-represented in arrests and referrals for status offenses, family offenses, and alcohol and drug offenses, they are not over-represented in arrests and referrals for most violent and property offenses.^8^ While both pathways from trauma to delinquency are important, the first may be especially relevant for Native youth.

Given the significance of trauma as a driver of delinquency for Native youth, any program that serves them must grapple with the question of what constitutes a trauma-informed intervention. Yet, few medical researchers have specifically examined effective approaches for treating trauma in Native youth, and few social science researchers have examined whether particular programs or approaches actually deliver state of the art trauma-informed treatment in the context of delinquency. While tribal justice systems have created innovative approaches for addressing trauma, existing research fails to link Indigenous approaches for addressing trauma with Western clinical research on trauma and trauma-informed approaches.

This commentary considers trauma-informed care for Native youth. It examines and contrasts the development and implementation of trauma-informed approaches in state and tribal juvenile delinquency systems. Nationally, about half of all Native youth who enter the delinquency system are under exclusive state jurisdiction, although some may be eligible for diversion to tribal systems.^9^ Youth living on reservations are under the jurisdiction of tribal delinquency systems. This jurisdiction is sometimes exclusive and sometimes concurrent with federal or state court jurisdiction.^10^ Overall, Native youth in state systems will usually encounter a model of care that was not developed with Native children or their communities in mind, even if the practices are billed as “trauma-informed” or “culturally competent.” Native youth in tribal systems may encounter a range of practices described as “trauma-informed,” ranging from clinical mental health treatment to community-based programs to cultural activities, most of which have not been modeled or tested.

In the clinical and social science literature, best practices for trauma-informed care are based on an abstract framework of trauma and trauma responses. This framework was not developed to address Native children’s needs but has nevertheless been applied to them. It is based on an individualized definition of trauma and an individual focus on processing traumatic experiences and building healthier coping mechanisms. Trauma-informed approaches to delinquency focus heavily on screening individual children and referring them to a separate mental health system to deliver specialized counseling, while leaving them formally involved (i.e., through probation or periodic incarceration) in a delinquency system that has not fundamentally changed. As further described below, such a system may further traumatize children.

Tribal systems, on the other hand, have created their own versions of trauma-informed delinquency systems. These systems have been developed in less-than-ideal circumstances. There are not enough trained mental health providers in most tribal communities to deliver the individualized screening and therapeutic care that is the hallmark of Western models. Even if there were, medical and psychological models for treating trauma do not adequately reflect the experiences of Native youth and communities. In this context, tribes have innovated models that focus on re-envisioning the entire approach to youth misbehavior from one focused on punishment and individual rehabilitation to one focused on community responsibility and an understanding of trauma that encompasses community-level trauma and its relationship to ongoing structural disadvantage. Restrictions on available funding mean that these services are usually delivered through the juvenile delinquency system (as opposed to completely redirecting young people into a separate mental health or child welfare system), but the resulting approaches challenge basic assumptions about what a delinquency system is.

We do not mean to suggest that tribal approaches are perfect, but we believe they are the best available options when compared to state systems that retraumatize Native youth. We call for more research to support, review, and develop tribally-driven models for trauma-informed delinquency interventions. Our goal is to move beyond justice policy buzzwords to support innovative and effective strategies for protecting Native children.

## Trauma-Informed Care in the Delinquency Context: The Western Model

In 2014, in response to the growing realization that trauma affects virtually all persons with mental and substance use disorders, the Substance Abuse and Mental Health Administration (SAMHSA) sought to further the development of a trauma-informed approach in to state justice systems. The 2014-2015 Policy Academy-Action Network Initiative, sponsored by the John D. and Catherine T. MacArthur Foundation and SAMHSA, released a report titled, “Strengthening Our Future: Key Elements to Developing a Trauma-Informed Juvenile Justice Diversion Program for Youth with Behavioral Health Conditions.”^11^ The report provided strategies for embedding trauma-informed approaches into state diversion policies and practices. This section discusses the research on childhood trauma that was the basis for the recommendations and the specific recommendations for how to implement trauma-informed approaches in the delinquency context.

### Defining and Measuring Trauma

The National Childhood Traumatic Stress Network (NCTSN) defines a traumatic event as “a frightening, dangerous, or violent event that poses a threat to a child’s life or bodily integrity,” or that causes a child to witness a threat to a loved one.^12^ Trauma has traditionally been defined in terms of direct life experiences. For children, these include experiences such as physical and sexual abuse, separation from parents, and witnessing violence.^13^ When a person directly experiences a traumatic event, that they face an increased risk of depression, suicidal behavior, and substance abuse.^14^ They may also experience a post-traumatic stress response.^15^

Psychologists measure trauma in children using the ACEs, or Adverse Childhood Experiences, scale.^16^ This measurement identifies ten major sources of childhood trauma and assesses how many of them a child has experienced — the more trauma, the higher the ACEs score. The ACEs framework emphasizes traumatic experiences that directly affect the individual and occur within the individual’s immediate family.

The ACEs framework does not always recognize the impact of community or environmental stressors, such as witnessing violence in the community, experiencing high levels of police surveillance and interaction, living in areas of concentrated poverty, experiencing racism, or attending school in a disruptive or violent environment. However, the NCTSN defines community violence as a type of trauma,^17^ and a large body of psychological and sociological research has linked structural factors (e.g., racism, poverty, unstable housing) and social determinants of health (economic stability, education, social and community context, health and health care, and neighborhood and built environment) to trauma.^18^ This kind of chronic stress can also place people at risk for post-traumatic stress disorder.^19^

Native children score highly on measures of individual, present day trauma experiences.^20^ They are also likely to face chronic stressors.^21^ Furthermore, theoretical and genetic research on intergenerational trauma (discussed further below) suggests that Native children as a group should be presumed to be impacted by historical trauma, regardless of ACEs score, family circumstances, or even an individual’s knowledge of specific historical events. Researchers have linked the experience of trauma with PTSD, mental health problems, and substance abuse among Native youth.^22^

### Trauma-Informed Care for Youth in the Delinquency System

As a social problem, childhood trauma must be addressed at multiple levels. Oral and colleagues describe primary (reducing the incidence of childhood trauma through prevention and family support), secondary (immediate interventions to reduce the negative impact of trauma), and tertiary (treating and reducing the long-term consequences of trauma) strategies.^23^ While primary strategies are the most effective, most of the literature on childhood trauma focuses on secondary and tertiary strategies. That is, childhood trauma is a concern among those who treat children who have already experienced it. At the secondary stage, trauma-informed care emphasizes psychological first aid, an approach developed for disaster survivors.^24^ Because children in the delinquency system are often acting out in response to trauma, trauma-informed care in the juvenile delinquency context is a tertiary strategy.

In this context, trauma-informed care “involves validation and recognition of the effects of traumatic events, common coping strategies, and effective treatments.”^25^ Trauma-informed care is a relatively new concept in delinquency systems, and many widely used approaches to delinquency involve practices that can worsen trauma.^26^ Implementing trauma-informed care in this context will often require reforming the whole system: training personnel to recognize trauma, reducing or eliminating practices that might retraumatize, emphasizing safety and transparency in all aspects of the system, from architecture to staff interactions, and providing specialized services to individual youth to reduce the impacts of trauma.^27^ SAMHSA describes this system-wide approach using the “four Rs”: “all people at all levels of the organization or system have a basic *realization* about trauma” and understand its effects, they are able to *recognize* the signs of trauma and the system *responds* by employing a trauma-informed approach in all aspects of its functioning and *resists retraumatizing* by eliminating harmful practices.^28^ Realization and recognition are achieved primary through (1) hiring and/or training staff in the psychology and biology of trauma and (2) the use of proven screening tools to identify trauma in children who come into the system. Thus, a juvenile court program should screen for trauma, emphasize safety, reduce or eliminate retraumatizing practices, and train staff to recognize the signs of trauma.

At minimum, this should involve adopting and training designated staff to use effective screening tools; training all staff to understand the sources and effects of trauma and to recognize trauma-related behaviors; and eliminating elements and practices that retraumatize. For example, a trauma-informed approach to residential delinquency programs might eschew locked doors, cell-like architecture, and the use of isolation and restraints. Staff might be trained in de-escalation practices.

Responding effectively to trauma also means ensuring the delivery of “trauma specific services,” or clinical interventions proven effective to treat trauma in individuals,^29^ for system-involved youth. The specifics of trauma treatment can vary by context, modality, and client needs. Levenson describes trauma-informed social work practice as focused on safety, trust, collaboration, choice, and empowerment.^30^ Specific interventions used in delinquency programs include Trauma and Grief Components Therapy for Adolescents (TGCTA), Cognitive Processing Therapy (CPT), Trauma-Adapted Multidimensional Treatment Foster Care (TA-MTFC), the Attitudes Related to Trauma-Informed Care (ARTIC) questionnaire, the Think Trauma Curriculum, the Sanctuary Model, and Trauma Affect Regulation Guide for Education and Therapy (TARGET),^31^ cognitive behavior therapy,^32^ Trauma Recovery and Empowerment, and Seeking Safety.^33^

In general, these are therapeutic models administered by a mental health professionals trained in trauma, its effects, and specific treatment approaches. Effective individualized approaches to treat trauma therefore require medical and mental health professionals trained in evidence-based approaches for addressing complex needs. Without those people on staff, many delinquency programs refer children to other programs or providers for treatment. Trauma-informed treatment thus requires *either* highly trained mental health staff *or* a robust referral system that integrates mental health treatment with court-ordered programming.

The dominant model for trauma informed delinquency programs assumes multiple overlapping systems for delivering services and the presence of many types of trained professionals. Best practices focus on training juvenile delinquency personnel (probation and correctional officers) to identify and screen for trauma, then implementing a referral network to allow children in the delinquency system to obtain individualized services from trained personnel in other systems, such as social workers and psychiatrists. Certain fundamental aspects of the delinquency system remain unchanged, such as the emphasis on public safety through containment and control of children, and a system that rewards obedience and punishes minor transgressions. Children may be diverted out of the system temporarily or completely to receive services intended to help them heal, but the system itself is not reconceived, and many aspects of it, such as separation from family and community, locked doors and restraints, guard oversight, open showers, and occasional violent confrontations between staff and youth, may retraumatize children.

## Trauma-Informed Care in Tribal Juvenile Systems: Lessons and Innovations

Acknowledging and addressing trauma has also been an important theme in Native communities. Today, most tribal justice systems espouse a commitment to “trauma-informed care.” In tribal systems, this term may be applied to a range of interventions, from therapeutic practices described in the previous section to cultural activities to community sessions focused on trauma. The resulting programs are largely untested, and they raise questions for further research about fidelity and efficacy. While it is important for researchers to assess the effectiveness of tribal programs, this assessment will require new models for trauma-informed care developed in collaboration with tribal communities. Put simply, it is a mistake for researchers to ignore tribal innovations just because they do not mirror the Western models for treating trauma, or to measure tribal programs against Western benchmarks.

Tribes’ work in this area reveals three key themes that are largely absent from the Western model. First, tribal approaches are holistic in that they seek to redefine the role of the legal system with regard to children. Second, Native approaches emphasize collective trauma, usually through a focus on how “historical trauma” has affected communities, and the relationship between individual and collective healing and well-being. Third, the emphasis on culture and tradition in Native approaches to addressing trauma, and the specificity of historical trauma, mean that each program is locally specific and developed under the leadership of that community. By highlighting these themes, we hope to create a bridge between tribal and Western approaches to addressing trauma in the delinquency system. Our goal is to ensure that Native children have access to the best practices developed in both contexts.

### Structural and Holistic Approaches

The dominant approach to trauma-informed care in the Western juvenile delinquency context centers on screening and referral. In this model, the delinquency system is one among many systems serving youth. Its focus is on addressing wrongdoing and its primary actors are police, probation officers, and correctional personnel. Other systems, such as child welfare, health, and mental health focus on caretaking. Guidance on trauma-informed approaches therefore focuses on training delinquency system personnel to recognize and properly screen for trauma to determine which children are in need of specialized care, then referring those children to other systems to receive individualized care.

In tribal juvenile systems, a commitment to trauma-informed care means assuming that most Native court-involved adults, children, and adolescents have experienced trauma and designing or modifying tribal court and probation processes to facilitate treatment of trauma and to avoid retraumatizing them. This is partly a response to a lack of resources and partly an acknowledgement of the way trauma has permeated tribal communities and institutions.

One model common in tribal juvenile systems is the Healing to Wellness Court (wellness court). The wellness court model has been developed by Native communities specifically to serve Indigenous people.^34^ Wellness courts combine western substance use disorder and behavioral health treatment with culturally-tailored interventions — providing substance abuse treatment, mental health treatment, and culture. These courts “utilize a nonadversarial approach, integrating traditional concepts of healing and community involvement toward healing, rather than punishing, their addicted tribal members.”^35^ Wellness courts may serve adults, children, and/or families. While wellness courts are not the only model tribes use for non-punitive, culturally-specific delinquency interventions, they share certain fundamental features that are common to Indigenous justice practices.^36^ Because they are the most widely studied and replicated model, we focus on them as a way to contrast Western and tribal approaches.

Wellness courts, which integrate intensive supervision via court/probation/case management processes with intensive outpatient treatment for substance use and mental health disorders, go deeper than the referral model in that they provide and supervise the actual “care.” Wellness courts typically require a fast-tracked clinical screening and assessment process, including mental health assessments when warranted. Participants in wellness court (those criminal defendants, parents alleged to have maltreated a child, and/or juveniles admitted to the wellness court program) agree to move through a phased treatment/supervision program over a year or so. Each wellness court participant assists in the development of an individualized treatment plan and ultimately, a relapse/aftercare plan. Wellness courts are team-based, where each team selects the treatment providers and cultural aspects that will serve its participants. The wellness court phases, like state drug court phases, include additional requirements, such as random drug testing, attendance requirements for case management, treatment and court hearings, and what it takes to move from one phase to another and to graduate from the program. The treatment modalities and complementary services include counseling, individual therapy, process groups, cognitive behavioral groups, education groups, support groups (AA, NA, Al-Anon), family therapy, multifamily groups, and other support services like housing, anger management, parenting skills, and experiential, wilderness and adventure-based programming.^37^ Success in the phases is reinforced by the application of incentives and sanctions in wellness court status hearings. Instead of referring children to experts outside the delinquency system, the Wellness court model — if implemented successfully — brings treatment into the system.

### Collective and Historical Trauma

The dominant Western conception of trauma as an individual experience is inadequate to capture the effects of collective trauma and community stressors over time. The concept of “historical trauma” refers to accumulated events and stressors that affected entire communities. For individual children, it provides an additional dimension to measurements of present days traumatic experiences. It also provides an important answer to the question of *why* Native youth experience such high levels of trauma and violence during their lifetimes. Collective trauma and unresolved grief may translate into high rates of community violence and may reduce the protective mechanisms available to children in these communities.^38^ While relatively new to Western medical and psychological research, the concept of historical trauma is not new to Native communities, and they have developed sophisticated models for treating and healing historical trauma.

Brave Heart defines historical trauma as “cumulative emotional and psychological wounding, over the life span and across generations, emanating from massive group trauma experiences.”^39^ The “historical trauma response” is described as a constellation of features associated with a reaction to massive group trauma.”^40^ For Native people, this includes historical unresolved grief, which is “the profound unsettled bereavement resulting from cumulative devastating losses, compounded by the prohibition and interruption of Indigenous burial practices and ceremonies.”^41^ Prominent examples include the Lakota children of survivors of the Massacre at Wounded Knee and children of survivors of boarding schools — and their descendants. It is understood that these experiences vary in degree across tribal communities.

Trauma may result from specific events, such as the Holocaust, the Tutsi genocide, or the Kosovo war. Yehuda and colleagues first identified intergenerational trauma among children of Holocaust survivors.^42^ For Indigenous communities each direct event is understood to be part of an ongoing experience of land loss, violence, poverty, forced migration, sickness, starvation, child separation, and deliberate suppression of cultural and religious activities. In consultation with Indigenous communities in the Upper Midwest U.S. and Canada, sociologist Les Whitbeck developed a measurement tool called the Historical Loss Scale (HLS), which “assesses the frequency with which Indigenous individuals think about the losses to their culture, land, and people as a result of European colonization.”^43^ Unliked the trauma screening tools used in juvenile courts, which seek to quantify specific individual traumatic experiences and associated symptoms,^44^ the HLS examines the significance of past trauma for people in affected communities without attempting to quantify the objective amount or seriousness of the trauma. A second scale developed by Whitbeck and colleagues, called the Historical Loss Associated Symptoms scale, identifies “emotional responses that are triggered” by reminders of historical traumas and ties those negative emotions to the awareness of historical trauma.^45^

Drawing on research demonstrating high rates of trauma among the descendants of Holocaust survivors, Brave Heart and Whitbeck describe historical trauma as something that is transmitted intergenerationally through memory, stories, behaviors, and parenting styles.^46^ More recent research has suggested that its effects may be transmitted genetically as well. This research identifies specific genetic changes attributable to trauma.^47^ Trauma can alter the expression of genes, changes that may be transient or fixed. In animals, traumatic experiences in the prenatal and early childhood stages have been linked to changes in genes that regulate stress reactions and affect behavior, including those that influence serotonin, estrogen, and neurotrophin activity and receptivity.^48^ Some of these genetic effects have been linked to greater vulnerability to mental health and substance abuse problems. The changes can be transmitted through both maternal and paternal lines and may occur across several generations.^49^

Research on humans is limited,^50^ and research in Native populations nearly nonexistent, but researchers hypothesize that epigenetic changes explain the persistent effect of historical trauma on Native people in the U.S. and other settler nations. This new research may be especially significant for Native children who do not live in tribal communities (and would therefore be involved in state juvenile delinquency systems), as it suggests these children may also be affected by historical trauma even if they are unaware of specific past experiences because they do not have access to community memories and behaviors that are usually assumed to be the conduit. Moreover, these epigenetic changes can affect individuals’ ability to cope with individual stressors experienced during their lifetimes, complicating the question of how to help people process and recover from direct experiences with traumatic events.

One frequently discussed source of intergenerational trauma is the separation of Native children from their parents and communities through federal assimilationist boarding schools and policies supporting the adoption of Native children to white families.^51^ The boarding school experience is relatively recent in time (1880-1950s) and has impacted Native people across tribes and generations. It has been well-documented that the boarding school experience has not only caused ripples of trauma in the survivors’ generation and with their children, but it has negatively impacted Native family structure and functioning and parenting, and has given rise to substance abuse and family violence.^52^

The concept of historical trauma entails a recognition that individual well-being is linked to community well-being. Collective experience with traumatic events and external stressors can add to and compound the individualized experience of trauma that is the focus of the ACEs scale. But the reverse is also true: addressing trauma at the community level is an important aspect of individual resilience. Matheson and colleagues explain that “resilience is fostered when survivors of natural events are able to express and anticipate collective solidarity and cohesion, and to act cooperatively to draw on collective social support resources … In effect, intergenerational resilience can be derived from the symbiotic relationship to the land, encompassing physical and non-physical elements, such as the social, emotional, and spiritual aspects that provide the foundation of identity, social connectedness, and a sense of community and belonging.”^53^ Porter and colleagues describe a similar concept of “self-healing communities.”^54^

The idea that individual children’s well-being is linked to group well-being undergirds Indigenous approaches to child welfare and juvenile delinquency and has even been expressed in U.S. and international law regarding children.^55^ Tribal communities seeking to address the effects of historical trauma often begin by organizing the entire community in programs intended to acknowledge and describe the collective impact of trauma.^56^ One reasons these programs are so important is that they normalize the experience of trauma, helping individuals see that they are part of a larger shared story, not defective. This sense of shared identity and affiliation becomes a protective factor for the next generation. Simply discussing historical trauma can be a useful healing tool when it “frames lifespan trauma in the collective, historical context, which empowers Indigenous survivors of both communal and individual trauma by reducing the sense of stigma and isolation.”^57^ On the other hand, Whitbeck and colleagues also found that thinking about historical trauma is associated with emotional distress, including depression and anger.^58^ It would appear that learning more, thinking, and talking about historical trauma can be both distressing and therapeutic, depending on how it is managed.

Collective acknowledgement and discussion may also help identify specific qualities and strategies for better health and management of trauma. Matheson and colleagues emphasize that some Holocaust survivors showed worse health outcomes, but a second group lived longer and seemed to be “uniquely hardy.”^59^ If some members of a community respond to historical trauma exhibit greater resilience, this suggests that traumatizes communities may be sources of strength, not just sources of damage. For Native children, an association with a community that has survived individually and persisted as a group is an important protective factor.

Wellness courts build on the relationship between collective and individual healing by involving extended families, members of the community, elders, and traditional leaders in the work of healing young people. Tribal court programs that emphasize tradition are working to connect Native youth with their communities and to instill a sense of pride and knowledge. When they incorporate acknowledgement of historical trauma, they are linking young people to a history of community survival and resilience.

### Community-Specific Models

Efforts to incorporate trauma-informed care in juvenile delinquency systems typically focus on evidence-based practices (EBPs), which are treatment modalities shown in studies to effectively treat trauma. However, most EBPs for treating trauma in children have not been evaluated in Native communities. Gameon and colleagues report that, of the six EBPs for treating trauma (trauma-focused cognitive behavioral therapy, Seeking Safety, prolonged exposure therapy, cognitive processing therapy, eye-movement desensitization and reprocessing, and child-parent psychotherapy) only two ( trauma focused CBT and Seeking Safety) have been adapted and shown to be effective Native communities.^60^ While evidence from other communities suggests that Native children may benefit from these approaches, the approaches were not developed to respond to the needs of Native children or the unique circumstances of tribal juvenile delinquency program. In response to the lack of proven approaches for Native children and/or a lack of the staff and financial resources to implement those approaches that are proven, Wellness courts have innovated local solutions for addressing trauma, even where these interventions have not yet been deemed EBPs.

Some practices are adaptations of EBPs. For example, the Indian Country Child Trauma Center (ICCTC) at the University of Oklahoma Health Sciences Center worked with the NCTSN and the SAMHSA to develop, refine, disseminate and evaluate culturally relevant trauma intervention models for use with children and adolescents in Indian country.^61^ The following interventions have been adapted from existing evidence-based treatment approaches: (1) Honoring Children, Making Relatives (adapted Parent-Child Interaction Therapy (PCIT) — the clinical application of parenting techniques in a traditional framework that supports the emphasis that AI/AN culture places on honor, respect, extended family, instruction, modeling, and teachings); (2) Honoring Children, Respectful Ways (designed to honor AI/AN children and promote their self-respect while also promoting respect for others, elders, and all living things; given traumas of sexual abuse, physical abuse, and violence, overlaid with cultural and historical trauma); (3) Honoring Children, Honoring the Future (includes the American Indian Life Skills Development Curriculum (AILSDC) as the evidence-based suicide prevention clinical component); and (4) Honoring Children, Mending the Circle (adapted TF-CBT, which applies cognitive behavioral techniques to address trauma in children, using traditional frameworks and AI/AN beliefs in spiritual renewal). These interventions would be relevant to trauma care in juvenile and family wellness courts, but it is unclear how many courts have implemented them.

The underlying premise of these adaptations is that Native/tribal culture contains therapeutics relevant to the building and repair of healthy relationships and healthy parenting. The four ICCTC interventions are built on common and tribe-specific cultural elements. The ICCTC further recognizes that interventions must be practical and useful for dissemination in rural tribal communities that lack licensed professionals. The ICCTC adaptation process started with the identification of core concepts within existing EBTs. The ICCTC then identified relevant traditional teachings and concepts, e.g., traditions related to parenting, nurturing, and therapeutic practice. They also focused on traditional ways of teaching, learning, and cultural worldviews that explain individual behavior. Finally they engaged in an ongoing dialogue with EBT developers and Native cultural consultants to finalize interventions, training materials, implementation strategies, and protocols.

Other practices are simply local cultural requirements, events and activities — culturally tailored interventions — that are incorporated as part of the wellness court phase requirements. For example, some of the early wellness courts couched these as “cultural components,” “community support meetings,” “other treatment activities,” and as graduation ceremonies. Brave Heart and colleagues have highlighted traditional ceremonies and practices as a potentially important way to treat historical trauma among Native people.^62^

The Menominee Indian Tribe of Wisconsin and it’s Meni¯paniw (Wellness Court) Program, have a “cultural component” which requires participants to participate in seasonal cultural activities (Winter Roundhouse, Teaching Lodge, Sugar Camp, Sturgeon Feast, Ricing, and Culture Camp) to enhance cultural knowledge, to build self-awareness, to improve self-esteem, and to build community wellness.^63^ They also recognize participants’ successful completion of the program with a ceremony showcasing the participant’s journey where the participants wears a ribbon shirt and tells their story.^64^ The Cass County Leech Lake Band of Ojibwe Wellness Court requires its participants to attend “community support meetings.” These may include Wellbriety, Talking Circles, and Sweat Lodges.^65^ The Yavapai-Apache Family Health Court provides for “other treatment activities,” including tribal culture, language, or history classes, optional spiritual counseling, and optional traditional healing.^66^ The Navajo Nation’s D.A.N.A. program allows for the use of a spiritual advisor, spiritual counseling, Native American Church ceremonies, other traditional ceremonies, and sweat lodges.^67^ It also provides for participation in Navajo Peacemaking.^68^ The Little Traverse Bay Band of Odawa Indians Waabshki-Miigwan (White Feather) Program incorporates the Seven Grandfather Teachings curriculum as a way to teach values and to denote participant progress in the program.^69^

In the first evaluation of four tribal drug courts, evaluators characterized “cultural education” as including prayers, sage burning, sweats, talking circles, fasting, preparing for Sun Dance, learning tribal history, and learning how to build a canoe or to tan a deerskin.^70^ They also noted that traditional aspects were incorporated in graduation ceremonies where honor songs were sung for the participants and where graduates were given blankets.

The Yurok Tribe Wellness Court also provides for participation in Red Road, talking circles, and sweat lodges.^71^ However, the Yurok Tribe Wellness Court stands out in that it created what one might describe as a parallel educational, mentoring, skills-building, and ceremonial training system. It created a “Cultural Division,” with three primary areas: cultural engagement, cultural competencies and traditional skills, and traditional health and healing.^72^ In the area of cultural engagement, it has peer counselors who work with youth and who are individuals involved in ceremonial and/or cultural activities that demonstrate leadership skills. The activities in this first area include cultural awareness building, Yurok teachings, and instruction on traditional knowledge. In the area of cultural competencies/traditional skills, it has cultural advisors who are recognized cultural artisans, linguists, and other individuals with proficient skills and who have acquired knowledge of tribal customs and traditions. The activities in this second area include survival skills; construction of equipment, tools, and accoutrements; food gathering, production and processing; traditional hunting and fishing skills; ceremonial regalia making; language acquisition; cultural awareness building; and development of a positive self and tribal identity. In the area of traditional health/healing, it has traditional practitioners who are respected elders, dance leaders, ceremonial leaders, and cultural caregivers. The activities in this third area include the teaching of traditional laws and custom; culture and gender-specific lifeways; tribal ceremonies and protocol instruction; intergenerational guidance on personal, family, and socio-cultural wellbeing; and oversight of family mediation and reconciliation.

Another more recent type of wellness court is the tribal-state joint jurisdiction wellness court, particularly family drug courts that serves youth and their families in cases coming from the delinquency, dependency, and criminal justice systems. A contemporary example of such a court is the Shingle Springs Band of Miwok Indians Tribal Court and Superior Court of El Dorado County Joint Jurisdiction Court.^73^ This joint jurisdiction court, in its materials, explicitly recognizes the Miwok experience of cultural, historical, and intergenerational trauma “through centuries of exposure to racism, warfare, violence, and catastrophic disease.”^74^ Indeed, the program screens for historical trauma.^75^ It also commits to using “a teamwork approach to address the needs of program participants using a culture-specific, trauma-informed, strengths-based, and evidence-based approach.”^76^ Participants work with team members to create culturally-based service plans.^77^ They state, “culture is an important protective factor for … young people.”^78^ In addition to wrap around services, the program includes lengthy lists of cultural activities and classes appropriate to the season of the year. Some of the learning objectives include historical-lateral trauma and grieving, forgiving, healing, and thriving, and motherhood and fatherhood are sacred.^79^

We note that simply incorporating elements of culture and tradition should not be conflated with adapting a comprehensive model of trauma informed care, let alone one that evidence based. For example, a state juvenile correctional facility that brings in Native elders to lead a sweat lodge ceremony is not necessarily implementing trauma-informed care. Nevertheless, when cultural practices are integrated into a system that involves the entire community and is designed with the goal of addressing youth trauma by strengthening identity and community resilience through traditional knowledge and practices, the resulting approaches — while most often untested — reflect many aspects of the academic and clinical research on addressing historical and intergenerational trauma. In this sense, Western research is just beginning to recognize the importance of group identification, cultural teachings, and traditional activities as a bulwark against the harms of trauma — ideas that Native communities have insisted upon for many decades.

While tribal justice systems have long incorporated traditional approaches that are described as restorative and trauma-informed, it is important to probe beyond these labels to determine how various approaches help or harm children. For example, some tribal detention programs incorporate cultural activities and traditional construction elements, but the use of locked doors, surveillance and restraints does not reflect accepted understandings of trauma-informed care for children. Similarly, programs that treat abuse by reintegrating the abuser in ceremonies or cultural activities may risk retraumatizing that abuser’s victims who live in the same community.

There is extensive debate about what should qualify as an EBT and how much EBTs can be adapted without losing the core aspects that have been demonstrated as being effective. Even when a model has been proven effective, questions remain about its implementation: is a particular community implementing the model with fidelity? Are there ways to improve on a tested model by making it more responsive to local needs? While it is important to evaluate EBTs developed in other communities and make helpful practices available to children in tribal communities, replication of Western models in tribal communities will always carry some problems. The historical trauma experienced by Indigenous communities is so specific, and research has established that community cohesion and culturally specific teachings are important in addressing it. This suggests that a practice should not be considered an EBT for Native children unless it has local involvement and is targeted to local problems. It is therefore important to devote research and program design resources to supporting, evaluating, and refining those “promising approaches” that are already in use in tribal juvenile systems, such as Healing to Wellness Courts. When a tribally-created model appears effective for other tribal communities, research should focus on how best to reproduce that model across communities in a way that is locally tailored but retains the core elements of the approach.

## Areas for Further Research

Developing and modeling effective trauma-informed interventions for Native youth will require expanded and sustained research in multiple fields.

Clinical researchers who study effective treatment for trauma should specifically consider which approaches work best for Indigenous children. This may entail research on whether existing models for trauma-informed care are effective for Native children and looking to tribal courts for innovate treatment models. It may also require developing new definitions of trauma and resilience and identifying protective factors unique to Native youth. For example, researchers should expand their focus from individual to community. Specifically:

- More medical and psychological research is needed on how Native youth experience and respond to trauma. Researchers studying historical and intergenerational trauma should study how this form of trauma affects Native people, whether specific historical experiences — such as boarding schools — result in different levels of inherited trauma, and how this form of trauma interacts with the experience of present-day ACEs.
- Indigenous researchers and tribal professionals should take the lead on this research, which will mean a need for more Indigenous medical and psychological professionals and better collaboration with tribal communities.
- Research should focus on specific tribes, be produced collaboratively with tribal governments, and thoughtfully incorporate traditional knowledge from that community. Partnerships between universities and tribes can support research to identify promising practices of local cultural origin, adapt models based on those practices, train system personnel on those models, and monitor the fidelity of the treatment modality to these models.
- Medical professionals working in tribal communities must be familiar with approaches developed by and for Native people and should employ those approaches before importing models developed outside Native communities.
- Researchers should study the effectiveness of wellness courts in delivering trauma-informed care. The question of effectiveness should be based on an expanded model, one that is developed by experts with knowledge about Native children and communities and in collaboration with the tribal community being studied. It should also consider whether and to what extent Wellness courts are delivering state of the art individualized care. Do they offer care that addresses the causes of anxiety, depression, PTSD, and addiction? Do they help youth identify and build upon protective factors? Are there aspects of the structure (e.g., sanctions or court or detention practices) that retraumatize children?
- State and local (non-tribal) delinquency systems should incorporate state of the art medical research when designing delinquency programs that serve Native youth. By “state of the art,” we mean both general medical research on trauma treatment and research on trauma in Native communities. Too often, professionals who design interventions for or work directly with Native youth are familiar with one area (either tribal approaches to historical trauma or Western approaches to individual trauma), but not the other.
- Tribal delinquency systems, including Healing to Wellness Courts, should consider adopting culturally adapted EBPs, such as those developed by the ICCTC, for individualized treatment. Where possible, they should hire or train qualified staff (including the use of telemedicine) to ensure that they can offer state of the art mental health and substance abuse counseling. Such counseling should be integrated with, not replace, cultural and community programming.
- Quality research will require sustained funding. Lack of funding is a longstanding problem for tribal courts, but here we believe it is possible to leverage the widespread interest in trauma-informed care and tribes’ status as leaders in restorative justice to generate research funding for tribal justice systems.

## Note

The authors have no conflicts to disclose.
